# Oct4 regulates the embryonic axis and coordinates exit from pluripotency and germ layer specification in the mouse embryo

**DOI:** 10.1242/dev.159103

**Published:** 2018-06-18

**Authors:** Carla Mulas, Gloryn Chia, Kenneth Alan Jones, Andrew Christopher Hodgson, Giuliano Giuseppe Stirparo, Jennifer Nichols

**Affiliations:** 1 Wellcome Trust–Medical Research Council Cambridge Stem Cell Institute, University of Cambridge, Tennis Court Road, Cambridge CB2 1QR, UK; 2 Department of Physiology, Development and Neuroscience, University of Cambridge, Downing Street, Cambridge CB2 4BG, UK

**Keywords:** Oct4, Epiblast, Lineage specification, Gastrulation, Pluripotency, Embryonic axis

## Abstract

Lineage segregation in the mouse embryo is a finely controlled process dependent upon coordination of signalling pathways and transcriptional responses. Here we employ a conditional deletion system to investigate embryonic patterning and lineage specification in response to loss of Oct4. We first observe ectopic expression of Nanog in Oct4-negative postimplantation epiblast cells. The expression domains of lineage markers are subsequently disrupted. Definitive endoderm expands at the expense of mesoderm; the anterior-posterior axis is positioned more distally and an ectopic posterior-like domain appears anteriorly, suggesting a role for Oct4 in maintaining the embryonic axis. Although primitive streak forms in the presumptive proximal-posterior region, epithelial-to-mesenchymal transition is impeded by an increase of E-cadherin, leading to complete tissue disorganisation and failure to generate germ layers. In explant and *in vitro* differentiation assays, Oct4 mutants also show upregulation of E-cadherin and Foxa2, suggesting a cell-autonomous phenotype. We confirm requirement for Oct4 in self-renewal of postimplantation epiblast *ex vivo*. Our results indicate a role for Oct4 in orchestrating multiple fates and enabling expansion, correct patterning and lineage choice in the postimplantation epiblast.

## Abstract (precis)

**Highlighted Article:** Ectopic expression of Nanog, expansion of the presumptive endodermal domain and trapping of cells in the primitive streak ultimately result in consistent disrupted phenotype with loss of embryonic structures.

## INTRODUCTION

How cells transition from pluripotency to differentiation is an important issue in developmental biology and the fundamental research that feeds into regenerative medicine. Lineage specification occurs in the epiblast of the early postimplantation mouse embryo in response to localised signalling cues, leading to acquisition of new cell identities and the instigation of body patterning. These instructive pathways emanate from a complex interplay of embryonic and extra-embryonic tissues. The process begins shortly after implantation, when a small number of cells in the distal visceral endoderm (DVE) acquires distinct molecular and morphological properties in response to Nodal signalling from the epiblast and bone morphogenetic protein (BMP) from the extra-embryonic ectoderm (ExE) (Arnold and Robertson, 2009; Brennan et al., 2001; Waldrip et al., 1998; Winnier et al., 1995). Through a combination of movement and additional cell recruitment, this population expands asymmetrically to become the anterior visceral endoderm (AVE). The main role of the AVE is to establish the anterior-posterior (A-P) body axis by secreting pathway inhibitors such as lefty, cerberus-like 1 (Cer1) and dickkopf-related protein 1 (Dkk1)*,* which negatively regulate primitive streak-inducing signals and restrict their activity to the posterior epiblast (Mukhopadhyay et al., 2001; Perea-Gomez et al., 2002). Conversely, the ExE dictates the proximal-distal axis (Rodriguez et al., 2005). Thus, the AVE and ExE set up a coordinate system interpreted by the epiblast, which leads to lineage specification *in vivo*. Genetic evidence suggests roles for various transcription factors in directing cell fate decisions in collaboration with the signalling pathways. However, the process by which multiple fates are coordinated in parallel is less well understood. This is largely because of the complex and relatively inaccessible three-dimensional and dynamic nature of gastrulation in murine embryos.

The availability of embryonic stem cells (ESCs) has enabled detailed investigations of exit from pluripotency and differentiation *in vitro* to be carried out, and provides a means with which to identify and assess the role of candidate factors. The POU domain transcription factor Oct4 (also known as Oct3 or Oct3/4) (Okamoto et al., 1990; Rosner et al., 1990; Scholer et al., 1990) has been proposed as an essential coordinating factor for pluripotent stem cell maintenance *in vitro* and for both preimplantation and postimplantation development *in vivo*. During the first cell fate decision in the preimplantation embryo, Oct4 prevents ectopic differentiation of the inner cell mass (ICM) into trophoblast, a phenotype mirrored in ESCs (Nichols et al., 1998; Niwa et al., 2000). Moreover, it also plays a non-cell-autonomous role in formation of the second extra-embryonic tissue, the primitive endoderm (PrE), which is attributable to induction of fibroblast growth factor 4 (FGF4) secretion by the epiblast (Frum et al., 2013; Le Bin et al., 2014). It has been postulated that Oct4 acts during lineage specification to induce ‘mesoendoderm’ fates (Thomson et al., 2011; Wang et al., 2012; Ying et al., 2015) and to repress neural differentiation (Thomson et al., 2011). Interestingly, Oct4 also plays a role in facilitating exit from pluripotency *in vitro* (Karwacki-Neisius et al., 2013; Radzisheuskaya et al., 2013).

Expression of *Oct4* (*Pou5f1*) persists in the murine epiblast until late gastrulation stages (Rosner et al., 1990; Scholer et al., 1990). Deletion at around embryonic day (E) 7.5 in a tamoxifen-inducible system caused developmental defects that were attributed to impaired expansion of the primitive streak (DeVeale et al., 2013). The authors also noted more severe defects in E9.5 embryos when *Oct4* was deleted at around E6 and E6.5. However, detailed analysis of the changes associated with loss of Oct4 during earlier stages of gastrulation has not yet been reported.

In light of these previous findings, we anticipated that disrupting *Oct4* expression during early postimplantation stages of development would provide a system with which to explore further the process of lineage specification *in vivo*. In this study, we used a compound conditional genetic model to delete *Oct4* from epiblast cells soon after implantation. We revealed an unexpected role for Oct4 in the regulation of both lineage identity and orientation of the A-P axis, and also confirmed its requirement for maintenance of postimplantation (‘primed’) pluripotency (Nichols and Smith, 2009). Furthermore, we applied a recently developed *in vitro* micropattern system (Morgani et al., 2018) to confirm our findings and enable dissection of lineage decisions during exit from pluripotency in an accessible two-dimensional format by inducible deletion of *Oct4*.

## RESULTS

### Deletion of *Oct4* during the onset of gastrulation causes reproducible disorganisation of epiblast derivatives

**Fig. 1.**
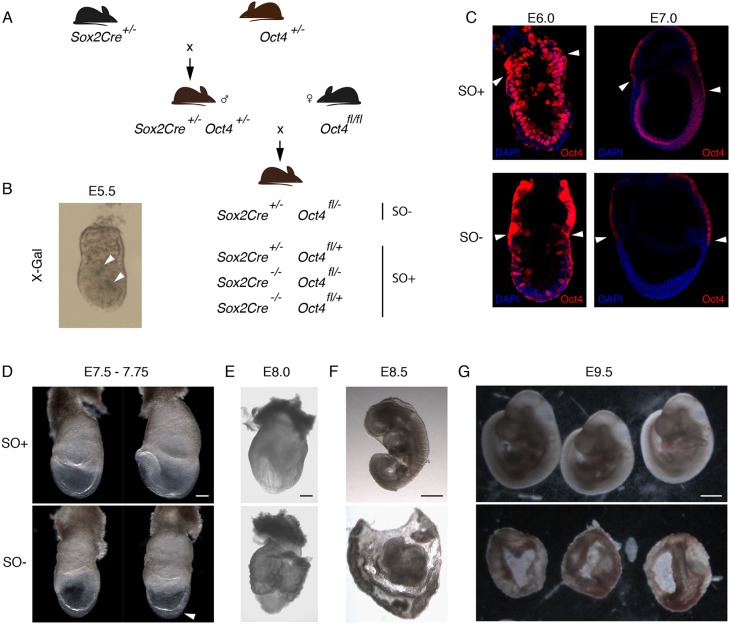
***Oct4* deletion strategy and phenotype.** (A) Schematic of mouse breeding programme to obtain conditional *Oct4* deleted (SO−) and control (SO+) embryos. (B) X-Gal staining of representative E5.5 embryos generated by crossing *Sox2Cre* and *R26-lacZ* homozygous mice, showing initial activation of the Sox2Cre in a few cells (blue staining, arrowheads). (C) Confocal images of immunostaining for Oct4 (red) in SO− embryos showing mosaic deletion at E6.0 and loss of Oct4 protein by E7.0. Arrowheads mark non-specific cytoplasmic staining in the extra-embryonic tissues. Embryo morphology is shown at (D) E7.5-7.75 (*n*=22 for SO−), (E) E8.0 (*n*=2 for SO−), (F) E8.5 (*n*=15 for SO−) and (G) E9.5 (*n*=3 for SO−). The top row in each panel displays control embryos and the bottom row contains mutants. The arrowhead in D indicates the thickened region in the mutant embryo, implying trapped cells in the primitive streak. Scale bars: 200 µm in D,E; 300 µm in F,G.

Transgenic embryos expressing *Cre* driven by the promoter of *Sox2* [Tg(Sox2-cre)1Amc; *Sox2Cre*] induce recombination of *LoxP* sites predominantly in epiblast cells soon after implantation (Hayashi et al., 2002) (Fig. 1A). We validated *LoxP* recombination by inspection of embryos derived from crossing *Sox2Cre* and *R26-lacZ* homozygous mice (Soriano, 1999). *Cre* activity was first apparent in a small proportion of cells at E5.5 (Fig. 1B), corroborating the zygotically driven embryonic expression of *Sox2* previously reported by mRNA *in situ* hybridisation (Avilion et al., 2003). To assess the consequence of *Oct4* deletion using this strategy, females homozygous for floxed *Oct4* (*Oct4^fl/fl^*) (Le Bin et al., 2014) were mated to males carrying *Sox2Cre* and an *Oct4*-null allele (Nichols et al., 1998) (*Sox2Cre/+; Oct4^+/−^*). Hence, we expect 25% of progeny to carry one *Sox2Cre* allele with one floxed and one null *Oct4* allele (*Sox2Cre^+/−^; Oct4^fl/−^*, referred to as SO−), which are classified as mutants. Of the remaining embryos, 50% are expected to be functionally heterozygous for *Oct4* (*Sox2Cre^+/−^; Oct4^fl/+^* or *Sox2Cre^−/−^; Oct4^fl/−^*) and 25% homozygous for functional *Oct4* (*Sox2Cre^−/−^; Oct4^fl/+^*), collectively referred to as SO+ (Fig. 1A). Immunohistochemistry revealed mosaic residual expression of Oct4 at around E6.0-6.5 and complete extinction by E7.0 (Fig. 1C). The first defects were apparent at around E7.5-7.75; the primitive streak appeared swollen and there were no discernible anterior structures (Fig. 1D and Table 1). By E8.0, the absence of anterior and trunk structures was more pronounced, and the remaining embryonic tissue was enveloped by extensively folded yolk sac (Fig. 1E). At E8.5, SO− epiblasts lacked headfolds, somites or any recognisable structures, and the embryonic regions were considerably smaller than those of SO+ littermates (Fig. 1F and Table 1). Only disorganised membranes remained by E9.5 (Fig. 1G). The striking morphological abnormalities confirmed the previously reported requirement for Oct4 during gastrulation (DeVeale et al., 2013). This consistent phenotype (Table 1) enables faithful examination of potential downstream lineage anomalies.

**Table 1.**
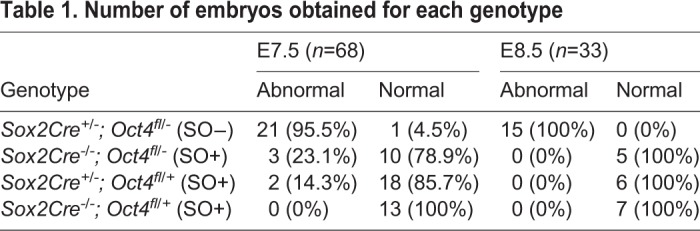
**Number of embryos obtained for each genotype**

### Oct4 mitigates expression of *Nanog* in the postimplantation embryo

**Fig. 2.**
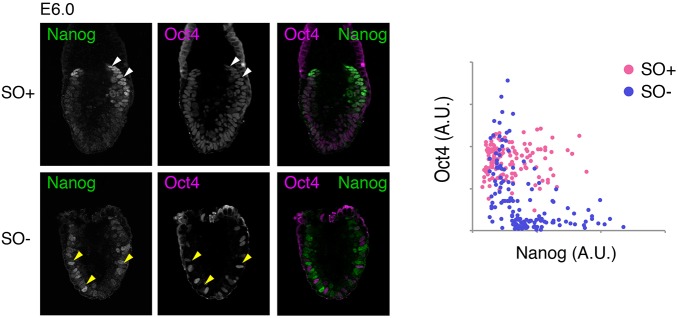
**Oct4 represses Nanog in the postimplantation epiblast.** (Left) Single confocal plane images of SO+ and SO− embryos immunostained for Nanog (green) and Oct4 (magenta) at E6.0 as Oct4 is being deleted. Arrowheads highlight regions of mutually exclusive expression of both markers. (Right) Quantification of immunofluorescence per cell (*n*=3, SO−; *n*=2, SO+ embryos).

Re-emergence of *Nanog* expression in the posterior proximal epiblast pre-empts the morphological manifestation of the primitive streak (Hart et al., 2004). We have previously shown that deletion of *Oct4* in the preimplantation epiblast results in upregulation of Nanog (Le Bin et al., 2014). We therefore analysed Nanog expression by immunohistochemistry in embryos dissected at E6.0-6.5, at the time when *Oct4* deletion is mosaic (Fig. 1C). In SO+ embryos, we observed uniform distribution of Oct4 throughout the epiblast, with Nanog present in only a proportion of cells in the proximal posterior region (Fig. 2). In contrast, SO− embryos exhibited mutually exclusive distribution of Nanog and Oct4 in an apparent ‘salt and pepper’ pattern throughout the epiblast, both posteriorly and anteriorly, where Nanog is not normally detected (Fig. 2). These results suggest that a cell-autonomous role for Oct4 in regulating Nanog is conserved in both pre- and postimplantation epiblast.

### Loss of Oct4 disrupts the axial organisation of emerging lineages

**Fig. 3.**
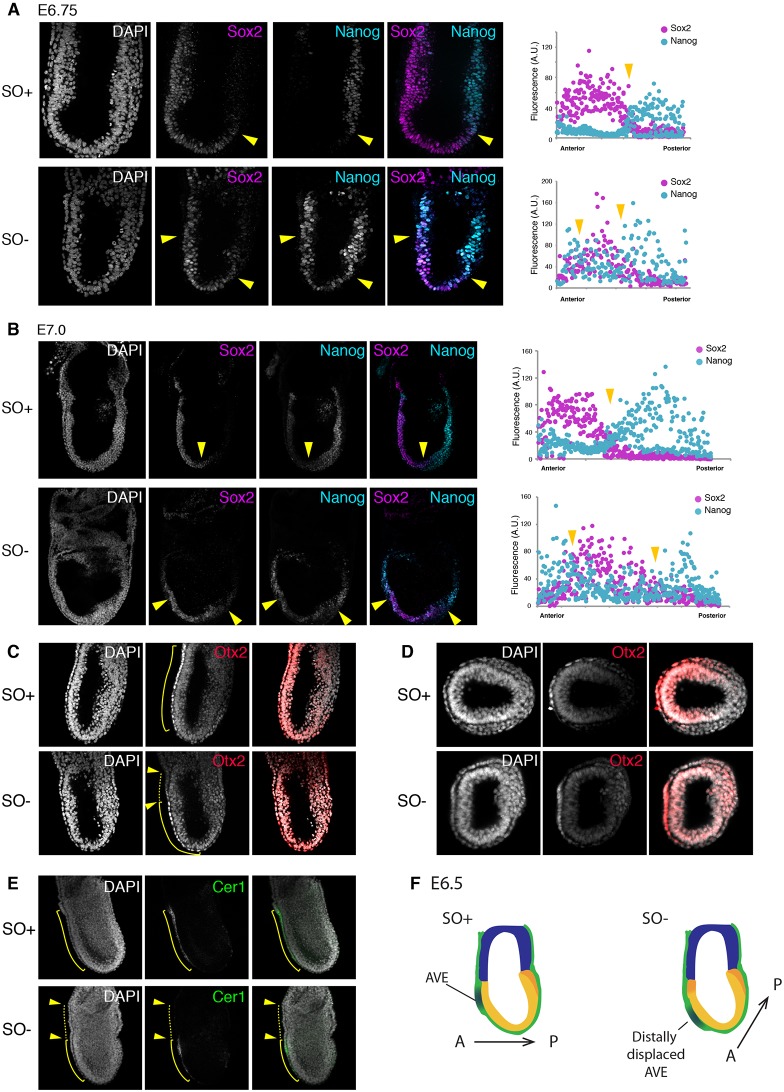
**In the absence of Oct4, domains of anteriorly expressed genes are distally displaced.** (A,B) Confocal images of immunostaining for Nanog (turquoise) and Sox2 (magenta) at E6.75 (A) and E7.0 (B) with DAPI in the left-hand panel. Single-cell immunofluorescence intensity quantification is shown in the graphs, as measured along A-P axis. Yellow arrowheads indicate the boundaries of expression domains between Nanog and Sox2. (C,D) Immunostaining of Otx2 (red) at E7.0 in whole-mount (C) and transverse (D) sections. (E) Expression of Cer1 in the AVE in SO+ (top) and SO− (bottom) embryos. In C and E, the yellow line indicates the region of Otx2-high and Cer1 expression in the VE. Yellow arrowheads indicate an Otx2-low and Cer1-negative domain in the anterior-proximal VE of SO− embryo. (F) Summary diagram of the differences between control and mutant embryos. At E6.5, control embryos initiate the expression of primitive-streak markers (orange) at the proximal-posterior portion of the epiblast (yellow), opposite to where the AVE is specified. In Oct4-deleted embryos, however, the AVE positioning is more distal, and ectopic Nanog expression appears anteriorly.

As Nanog is ectopically expressed in the anterior epiblast in SO− embryos, we sought to determine whether the expression pattern of specific anterior genes was disrupted. At E6.75 and E7.0, Sox2 normally segregates to the anterior epiblast in a pattern complementary to Nanog (Fig. 3A,B). However, in mutant embryos, the boundaries of Nanog and Sox2 domains were less defined. The Sox2 territory appeared to be shifted distally and the Nanog domain expanded (Fig. 3A,B). Interestingly, we also observed the appearance of an ectopic Nanog-high domain, anterior to that of Sox2.

We also examined the expression pattern of Otx2, which functions both in epiblast and visceral endoderm (VE) (Ang et al., 1996; Perea-Gomez et al., 2001). Otx2 is initially expressed throughout the epiblast and VE but during gastrulation it becomes restricted anteriorly (Ang et al., 1994) (Fig. 3C,D). This pattern was not observed in mutant embryos (Fig. 3C,D). Moreover, although SO+ embryos showed high expression of Otx2 in the AVE, in the mutants the Otx2-high domain was displaced distally (Fig. 3C, yellow line) and an Otx2-low domain appeared in the anterior-proximal VE (Fig. 3C, yellow arrows). We therefore immunostained for Cer1, a specific marker of the AVE. Like Otx2, Cer1 expression was more distal in SO− embryos compared with SO+ (Fig. 3E). These observations suggest that, in the absence of Oct4, embryos fail to maintain correct A-P orientation, resulting in an apparently more distal positioning of the body axis (Fig. 3F).

### Oct4 restricts the expression of definitive endoderm transcription factors

**Fig. 4.**
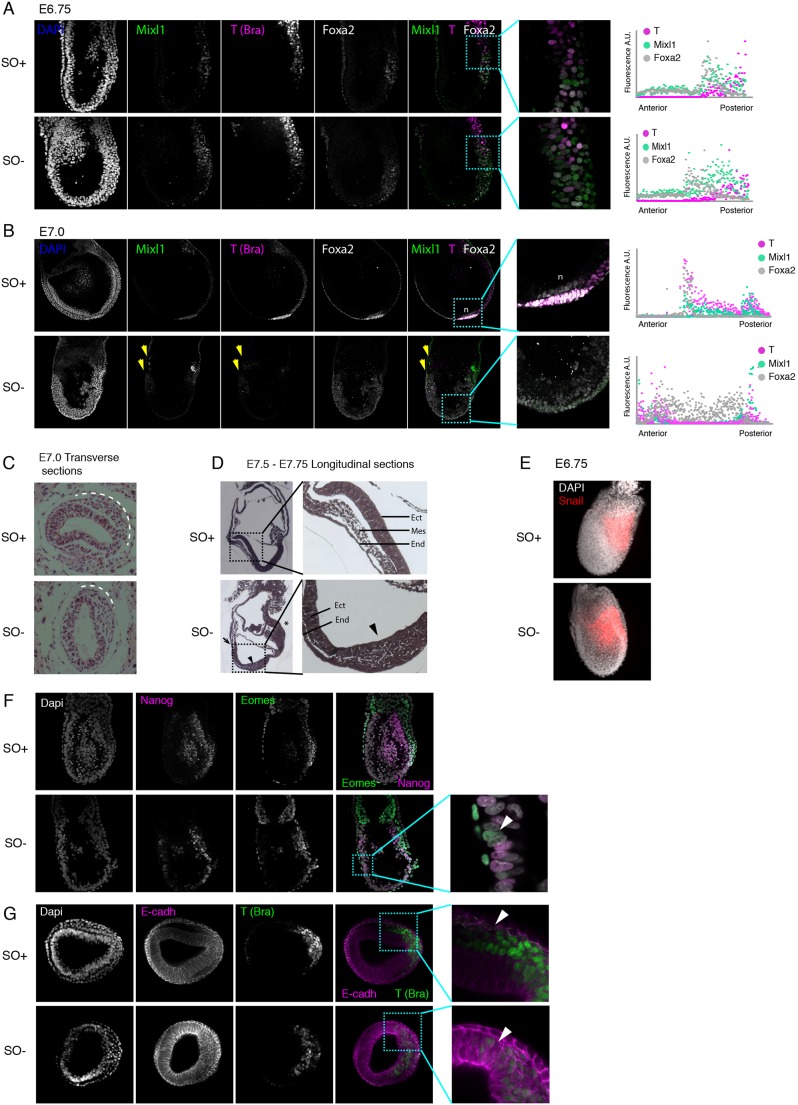
**Ectopic expression of posterior genes in *Oct4* mutant embryos.** (A,B) Confocal images of immunostaining for Mixl1 (green), T (brachyury, magenta) and Foxa2 (white) in E6.75 (A) and E7.0 (B) embryos. Arrowheads indicate the boundaries of the ectopic posterior-like domain. Graphs show quantification of the expression domain along the A-P axis. (C) Transverse section and H&E staining of SO+ and SO− embryos at E7.0. Dotted line highlights the extent of the mesoderm migration from the middle of the streak. (D) Longitudinal sections and H&E staining of embryos at E7.5-7.75. Expanded view shows three germ layers in SO+ embryos. Asterisk highlights the accumulation of cells in the extra-embryonic region; the arrow shows a lack of anterior structures and arrowheads mark accumulation of cells in the posterior epiblast. (E) Confocal images showing immunostaining of Snail (red) in SO+ and SO− embryos at E6.75. (F) Confocal images of Eomes (green) and Nanog (magenta) double immunostaining. Arrowhead in the enlarged view points to an Eomes-positive cell in the anterior epiblast. (G) Transverse sections of E7.0 embryos immunostained for E-cadherin (magenta) and T (brachyury, green) in SO+ and SO− embryos. Enlarged views and arrowheads indicate downregulation of E-cadherin and T in migrating mesoderm in SO+ embryos, but not in SO− embryos. Top row of panels in each case shows SO+ embryos and bottom panels show SO− *Oct4* mutants.

The expression pattern of proteins associated with the primitive streak was analysed. At E6.75, before any overt morphological phenotype is detected in mutant embryos, the pan-primitive streak markers brachyury (T) (Wilkinson et al., 1990) and Mixl1 (Pearce and Evans, 1999; Robb et al., 2000) were detected exclusively in the posterior epiblast (Fig. 4A). Similarly, Foxa2 was expressed in a posterior-distal domain. Foxa2 marks a group of mesoderm and endoderm progenitors that segregate after epithelial-to-mesenchymal transition (EMT) (Burtscher and Lickert, 2009) (Fig. 4A). The relative expression pattern of all three proteins was generally conserved between SO+ and SO− embryos at E6.75. Therefore, the primitive streak appears to be initially specified in the correct location in SO− embryos.

In E7.0 mutant embryos, the expression domain of T and Mixl1 appears to be significantly reduced (Fig. 4B). We also noted a second region expressing T and Mixl1 in the anterior epiblast (Fig. 4B, yellow arrows), coincident with the ectopic Nanog domain (Fig. 3). Interestingly, the expression domain of Foxa2 expanded dramatically both anteriorly and posteriorly through most of the epiblast, but was absent at the ExE-epiblast boundary (Fig. 4B). SO− embryos also lacked a distinguishable node (‘n’, Fig. 4B).

We examined the tissue histology in embryos by Haematoxylin and Eosin (H&E) staining of longitudinal and transverse sections. At E7.0, a layer of mesenchymal cells encases more than half the epithelial epiblast in SO+ embryos. In SO− embryos, however, loosely packed cells accumulate at the posterior end of the embryo (Fig. 4C). This is consistent with the apparent swelling in the mesoderm of mutants observed previously (Fig. 1D) and was more evident in longitudinal sections at E7.5-7.75. Mutant embryos show accumulation of cells and loss of epithelial morphology in the posterior epiblast (Fig. 4D, arrowhead). We also noticed cells amassing in the extra-embryonic region adjacent to the streak, and the absence of anterior structures (arrow) in SO− embryos (Fig. 4D).

During normal gastrulation, cells in the epiblast exhibit high levels of E-cadherin, which is downregulated as they traverse the primitive streak and undergo EMT. This downregulation in E-cadherin is brought about by the repressor snail (Carver et al., 2001). In SO+ and SO− embryos, snail expression was unaffected (Fig. 4E). The expression pattern of Eomes, another key regulator of EMT (Arnold et al., 2008), was also mostly unchanged upon Oct4 deletion (Fig. 4F). However, we noted a few Eomes-positive cells in the anterior epiblast (Fig. 4F, arrowhead). SO− embryos, however, show persistent E-cadherin expression in the cells surrounding the primitive streak, indicating an EMT defect (Fig. 4G).

To summarise, in SO− embryos the primitive streak is initially correctly specified, but by E7.0 the expression domain of Foxa2 extends both anteriorly and posteriorly at the expense of more proximal-posterior primitive streak markers. Furthermore, loss of Oct4 causes a defect in downregulation of E-cadherin, leading to failure to complete EMT and consequent retention of cells in the posterior epiblast.

### Disorganisation of Oct4 mutant epiblasts disrupts subsequent lineage differentiation

**Fig. 5.**
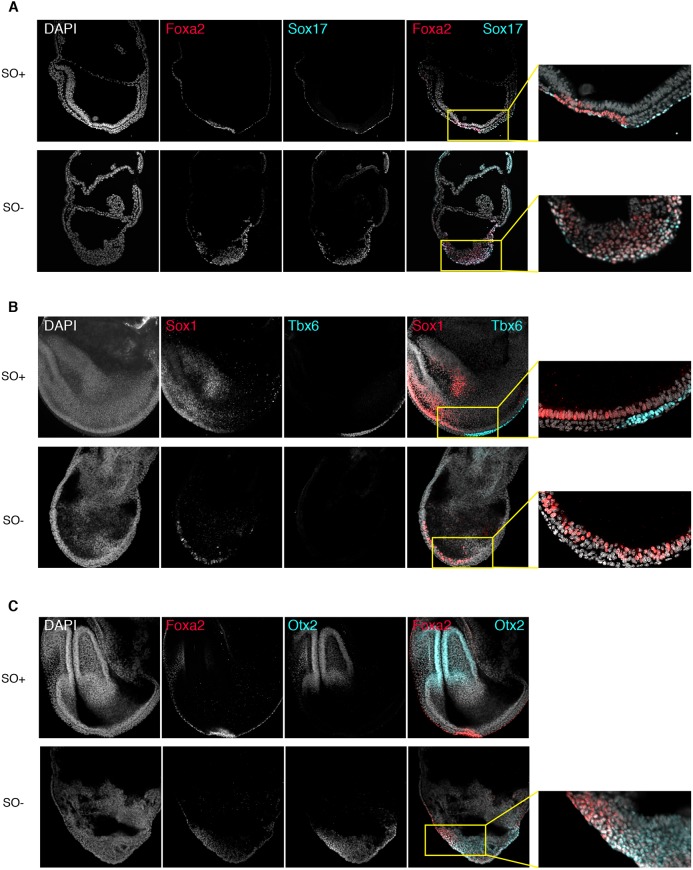
**Disorganisation of lineage markers in *Oct4* mutant embryos impedes further differentiation.** (A-C) Confocal images of immunostaining of E7.5-7.75 embryos for (A) Foxa2 (red) and Sox17 (turquoise), (B) Sox1 (red) and Tbx6 (turquoise), and (C) Foxa2 (red) and Otx2 (turquoise). Top rows show SO+ embryos and bottom rows show SO− *Oct4* mutants. Higher-magnification images show regions of marker segregation (A and C, digital enlargement; B, captured using a 40× lens).

In the absence of Oct4, embryos acquire disordered anterior and posterior gene expression patterns, and fail to complete EMT. We therefore examined the expression of germ layer markers expressed later in development. Sox17 is normally expressed in committed definitive endoderm cells (Viotti et al., 2014). Consistent with the expanded domain of Foxa2, we observed ectopic Sox17 in many cells of the anterior and posterior epiblast at E7.5, in a subpopulation overlapping the Foxa2-positive domain (Fig. 5A). To determine whether any anterior structures were specified, we analysed the expression of Sox1, a neuroepithelium marker (Fig. 5B) (Pevny et al., 1998). Despite the lack of clear anterior structures in SO− embryos (Figs 1D and 4D), Sox1 expression was detected, but more distally than in SO+ embryos and in a ‘salt and pepper’ fashion (Fig. 5B). Otx2, conversely, is normally expressed in the headfolds at E7.5 (Ang et al., 1994; Cajal et al., 2012), but in SO− embryos it was expressed in the distal epiblast in a domain overlapping Foxa2 (Fig. 5C).

We have previously detected expression of T and Mixl1 in both anterior and posterior epiblast in a small proximal domain of SO− embryos, suggesting Oct4 is not absolutely required for expression of these early markers of the primitive streak (Fig. 4B). Therefore, we examined Tbx6, a marker of paraxial mesoderm (Chapman et al., 1996; Chapman and Papaioannou, 1998) and thus an indicator of further differentiation. Tbx6 was not detected in SO− embryos, consistent with a defect in EMT and abrogation of embryonic mesoderm differentiation (Fig. 5B). To examine whether the defect in later-stage mesoderm differentiation results from defective EMT or indicates an absolute requirement for Oct4 in mesoderm differentiation, we carried out explant cultures of mutant embryos. Plating E7.5 embryonic tissues on a feeder layer of mitotically inactivated murine embryonic fibroblasts in basal N2B27 medium resulted in beating foci after 48 h, both in SO+ and mutant embryos (Movies 1 and 2). This suggests that the lack of mature mesoderm structures in Oct4 mutant embryos might arise from the defective tissue organisation, rather than from an absolute requirement for mesoderm specification. However, we cannot exclude a specific function for Oct4 in specifying more distal primitive streak derivatives.

The disruption in lineage segregation apparent in our SO− embryos provides an opportunity to investigate the potential compatibility rules for lineage-specific gene expression. We therefore ascertained whether deletion of *Oct4* could result in aberrant co-expression of lineage-specific transcription factors. Co-staining for Nanog and Sox1 revealed that although Nanog expression persisted throughout the epiblast of mutant embryos up to the headfold stage, it was mutually exclusive with Sox1 at the single-cell level (Fig. S1A). Similarly, despite the widespread upregulation of Sox17 in mutant embryos, Sox1 and Sox17 were never observed in the same cell (Fig. S1B). Therefore, lack of Oct4 results in widespread tissue disorganisation but apparently not in aberrant co-expression of genes that would normally be mutually exclusive.

We conclude that the defects observed in Oct4 mutants during early gastrulation severely affect subsequent attempts to establish more mature lineages. Although markers of advanced anterior differentiation are detected, their expression domain is more distal than in SO+ embryos, consistent with the lack of anterior structures. Markers of lineages that arise from the posterior primitive streak are missing, as expected from the posterior epiblast disorganisation and the failure of cells to undergo EMT.

### Lack of Oct4 results in broad mis-regulation of transcripts

**Fig. 6.**
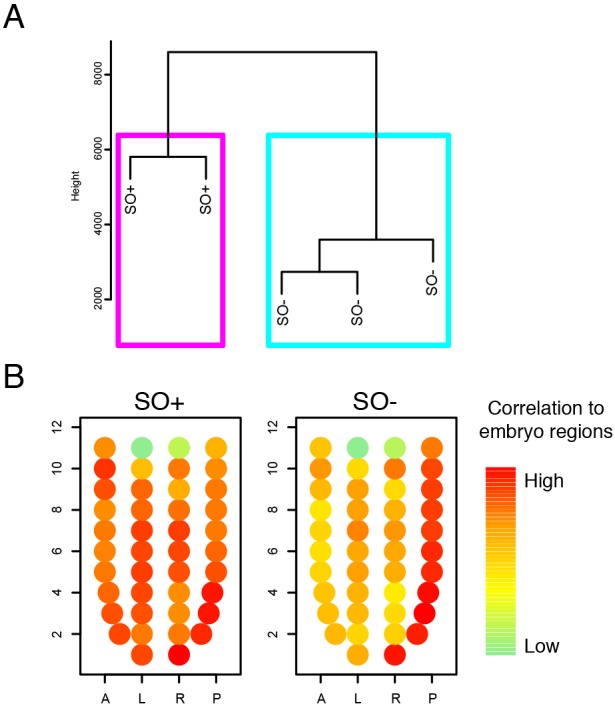
**Gene expression analysis of embryos.** (A) Hierarchical clustering of SO+ and SO− epiblasts and VE dissected from E6.75 embryos. (B) Correlation of SO+ and SO− samples with each embryo region shown as a circle, in its corresponding position: 1, distal; 11, proximal; A, anterior; P, posterior; L, left; R, right.

In order to identify the first changes associated with *Oct4* deletion, we carried out RNA-sequencing of whole epiblasts after *Oct4* deletion, but before any morphological changes were clearly evident, at ∼E6.75. Hierarchical clustering clearly separated SO+ and SO− embryos (Fig. 6A). Of the 372 differentially expressed transcripts (adjusted *P*-value<0.05), 240 were significantly upregulated and 134 were downregulated in the mutant embryos, compared with controls (Table S2). Global analysis of signalling pathways or gene ontology terms did not reveal any significant enrichment. Therefore, we examined the expression of signalling pathway components relevant to EMT and lineage specification (Fig. S2). Although some relevant pathway components were differentially expressed, no clear trend could be observed (Fig. S2). Furthermore, none of the growth factors, receptors or downstream targets varied in a manner consistent with the phenotype. For example, FGF8 and Fn1 proteins, which positively regulate EMT (de Almeida et al., 2016; Sun et al., 1999), were upregulated in SO− embryos. Overall, most pathways relevant to posterior epiblast identity were slightly higher in SO− embryos compared with SO+. This suggests that the SO− phenotype is caused by mis-regulation of key lineage specifiers rather than by upstream signalling pathways.

Self-renewing primed pluripotent stem cell lines, known as ‘epiblast stem cells’ (EpiSCs), can be captured by explanting postimplantation epiblasts (Brons et al., 2007; Tesar et al., 2007) and their identity resembles the gastrulation stage epiblast (Kojima et al., 2014). Therefore, EpiSCs provide a convenient *ex vivo* system with which to perform biochemical assays. We examined recently published Oct4 ChIP-seq data in EpiSCs (Matsuda et al., 2017). Most (239) differentially expressed genes showed Oct4 binding peaks within 50 kb of the gene promoter (Fig. S4A). We also identified Oct4 binding peaks upstream of Foxa2, Otx2 (Fig. S4B) and most components of the pathways relevant to EMT (Fig. S3). These findings are consistent with a potential role for Oct4 in regulating multiple fates.

Our immunohistological studies detected mis-expression of posterior epiblast genes (Fig. 5). To extend this analysis at a genome-wide level, we compared expression in SO+ and SO− embryos with the published spatially localised transcriptome data of mid-gastrulation embryos (Peng et al., 2016). For each embryo region, we assessed the degree of correlation with our control and mutant embryos. As expected, SO+ embryos showed high correlation across all embryo regions, consistent with an equal representation of genes in SO+ and reference embryos (Fig. 6B). Conversely, SO− embryos showed high correlation to regions of the distal/posterior epiblast, but much less with anterior and left/right regions (Fig. 6B).

Overall, this analysis provides no support for the hypothesis that the Oct4 phenotype results from dramatic switches in the activity of signalling pathways. Instead, it suggests that Oct4 orchestrates the underlying transcription factor network in a cell-autonomous manner.

### *Oct4* mutant phenotypes are not dependent upon disruption of upstream signalling pathways

**Fig. 7.**
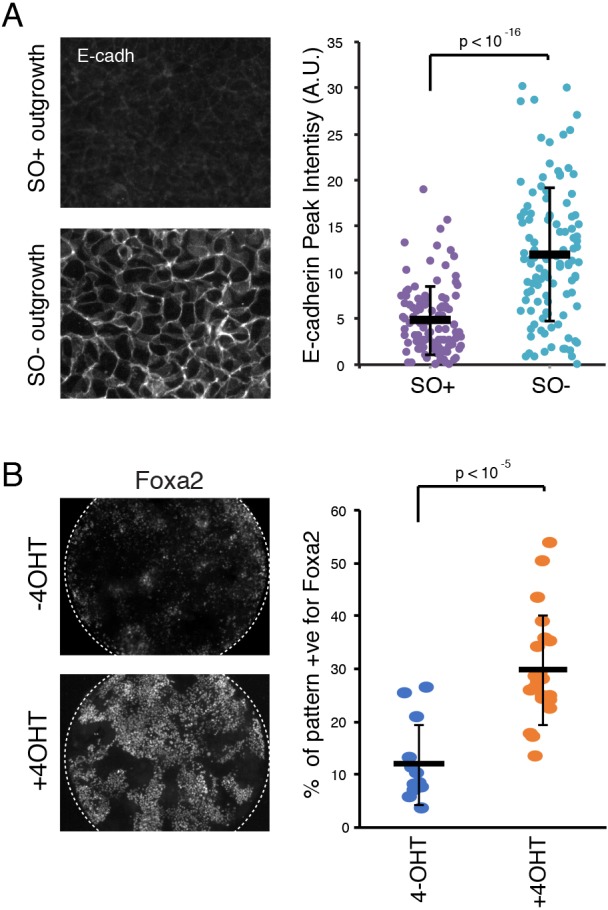
**Recapitulation of SO− phenotypes *in vitro*.** (A) Immunostaining and quantification of E-cadherin in SO+ and SO− epiblast outgrowths dissected at E6.5 and cultured for 48-72 h in N2B27. Data are mean±s.d. (B) Immunostaining and quantification of Foxa2 expression in IOD cells cultured in EpiLC media conditions in the presence (bottom) or absence (top) of tamoxifen (4-OHT). Data are mean±s.d.

To determine whether the SO− EMT phenotype is cell-autonomous, epiblasts were liberated from their extra-embryonic tissues at E6.5 and plated on fibronectin in basal medium lacking exogenous signalling components or cytokines. Explants were cultured for 48-72 h, fixed and processed for immunohistochemistry. SO− explants exhibited significantly higher E-cadherin expression compared with SO+ controls (Fig. 7A), suggesting that the EMT phenotype is cell-autonomous.

As an alternative approach, we made use of a recently developed *in vitro* culture system for patterning cells in culture (Morgani et al., 2018). Mouse ESCs were derived from homozygous compound transgenic embryos in which *Oct4* can be ubiquitously deleted in a tamoxifen (4-OHT)-inducible manner. Cells were first primed towards an epiblast-like cell (EpiLC) state (Hayashi et al., 2002; Morgani et al., 2018) before plating on to laminin-coated micropatterns. As shown previously in EpiLC conditions, control cells maintain expression of Oct4 and do not upregulate Foxa2 (Fig. 7B) (Morgani et al., 2018). In the presence of 4-OHT, however, we observed widespread upregulation of Foxa2, consistent with our *in vivo* data (Fig. 4B).

### Oct4 is essential for establishment and maintenance of EpiSCs

Our data so far imply an important role for Oct4 in choreographing postimplantation development. We therefore investigated whether it is required for maintenance of primed pluripotency in the context of self-renewal of epiblast cells *ex vivo*. EpiSC derivation efficiency is greatly enhanced by addition of the Wnt inhibitor XAV 929 (Sumi et al., 2013). We attempted to derive EpiSCs from E5.75 and E6.5 SO− and SO+ embryos. EpiSCs were readily obtained from SO+ epiblasts (23/23), but not from SO− epiblasts (0/11). As an alternative approach, we made use of homozygous compound transgenic embryos in which Oct4 may be deleted in a 4-OHT-inducible manner from inducible Oct4 deletion (IOD) embryos (Le Bin et al., 2014). EpiSCs were successfully derived from IOD embryos in the absence of 4-OHT (2/2), but not in its presence (0/8). This was unlikely to reflect toxicity issues from the inducing agent or activation of Cre, as EpiSCs were efficiently produced from Confetti (Snippert et al., 2010)×R26-CreERT2 (Vooijs et al., 2001) *inter se* crosses exposed to the same concentration of 4-OHT (14/14). Interestingly, trophoblast stem (TS) cells readily emerged from explants of SO− embryos explanted into the same culture conditions. These lines were morphologically identical to the TS cells obtained from explanted SO+ ExE, suggesting that the SO− TS cell lines were derived from ectopic ExE encroaching upon the defective epiblast. In confirmation of this, early passage SO− explants contained mostly cells that were negative for *lacZ*, the reporter for expression of the *Oct4*-null allele (Nichols et al., 1998), confirming their identity as extra-embryonic cells. Finally, we attempted to delete *Oct4* from previously established IOD EpiSC lines but found that upon addition of 4-OHT, no EpiSC lines could be passaged. We conclude that Oct4 is required for propagation and self-renewal of the primed state of pluripotency.

## DISCUSSION

We have used conditional deletion of *Oct4* in gastrulating mouse embryos to explore the mechanism of exit from pluripotency and progression to lineage specification *in vivo*. Activation of Cre recombinase by the *Sox2* promoter resulted in loss of Oct4 between E6.5 and E7.0, and consistent abnormalities causing embryonic lethality by E9.0 (Fig. 1). The time of Oct4 deletion suggests that the embryonic axis is initially correctly specified. At around E6.0, Oct4 protein was extinguished from epiblast cells in a mosaic, ‘salt and pepper’ pattern, leading to ectopic expression of Nanog in a manner complementary to Oct4 (Fig. 2). Upregulation of Nanog following *Oct4* deletion has been reported previously in preimplantation embryos (Le Bin et al., 2014). Our new results identify a cell-autonomous role for Oct4 in moderating Nanog expression in primed, as well as naïve, pluripotent embryonic cells (Nichols and Smith, 2009). When examining lineage specification, we found that embryos lacking Oct4 failed to maintain body axis orientation. Instead, the anterior domain becomes more distal and a second ‘posterior’-like domain appears in the anterior-proximal epiblast. This was determined by the more distal localisation of the expression domains of Sox2, Sox1, Otx2 and Cer1 (Figs 3 and 5), and by the appearance of a second anterior-proximal domain positive for Nanog, brachyury (T) and Mixl1 (Figs 3 and 4). Concomitantly, endoderm markers, which are normally expressed in the distal epiblast, became upregulated and their expression domains extended proximally, both in the anterior and posterior direction, although not as far as the proximal ExE-epiblast boundary. This expansion of endoderm markers coincided with reduced expression domains of proximal-posterior genes (T and Mixl1) as well as failure to upregulate more mature mesoderm markers, such as Tbx6, which denotes paraxial mesoderm. Examination of sections also revealed defects in cell morphogenesis. In *Oct4* mutant embryos, cells accumulated in the streak and failed to migrate out. Indeed, at E7.5, there were no distinguishable cell layers in the posterior epiblast and cells appear as a single disorganised mass (Figs 4 and 5). This is probably attributable to the observed failure to downregulate E-cadherin, or E-cadherin re-expression after *Oct4* deletion (Fig. 4). Interestingly, we observed that explant cultures were able to give rise to beating cardiomyocytes (Movies 1 and 2). This, as well as the initial expression of posterior primitive streak-markers T and Mixl1 after *Oct4* deletion, argues against direct requirement for Oct4 in specifying mesoderm. However, as all the markers detected are characteristic of the proximal primitive streak, it remains to be determined whether the defects in distal primitive streak specification are indirectly caused by the defects in EMT or because of a cell-autonomous role for Oct4 in specification of these fates.

As Oct4 is a transcription factor, a key question is which of its targets lead to the observed phenotype? Such abrupt changes in patterning could be due to two possible extreme mechanisms. First, deletion of Oct4 could result in changes to the signalling pathway landscape within the epiblast. This would result mostly in non-cell autonomous defects. Because Oct4 is not expressed in extra-embryonic tissues, the more distal positioning of the AVE in SO− mutants (Fig. 3C,E,F) is likely to be caused indirectly. This distal positioning in the mutants probably results in the appearance of a group of cells expressing primitive streak markers in the anterior-proximal epiblast. The ectopic expression of posterior-associated genes in the proximal-anterior epiblast in the SO− embryos is likely to account for the lack of anterior structures in later development (Fig. 4). However, transcriptome analysis of whole epiblasts did not detect differential gene expression enrichment in any signalling pathway, despite the fact that Oct4 binds widely in EpiSCs (Matsuda et al., 2017).

In contrast, deletion of *Oct4* could result in the mis-regulation of key transcription factors. Although Oct4 has been suggested to act primarily as a transcriptional activator for maintenance of pluripotency (Hammachi et al., 2012), it has also been reported to serve as a repressor in other contexts (Lang et al., 2009; Ouyang et al., 2009). The phenomenon of derepression of Nanog observed in Oct4-negative cells immediately after deletion of *Oct4* in the ICM (Le Bin et al., 2014) and postimplantation epiblast (Fig. 2) might extend to other genes, such as *Foxa2*. *Oct4* appears to bind upstream of *Foxa2* (Fig. S3B) and *in vitro* studies have suggested that Oct4 blocks Foxd3-mediated activation of *Foxa2* (Guo et al., 2002). In SO− mutants, the Foxa2 expression domain was greatly expanded (Fig. 4B), and Foxa2 was also upregulated in an *in vitro* differentiation assay upon inducible deletion of *Oct4* (Fig. 7B). Interestingly, Foxa2 is important for orchestrating multiple cellular identities. Forced expression of Foxa2 in E7.5 embryos results in posterior expansion of the Otx2 expression domain (Kimura-Yoshida et al., 2007), a phenotype we also observe upon *Oct4* deletion (Fig. 3C,D). Moreover, both Foxa2 and Otx2 are essential for maintaining correct A-P polarity via specification of the DVE; in the absence of either factor the body axis remains distal-proximal, so that primitive streak-associated genes such as *T* are expressed at the epiblast-ExE boundary and fail to become restricted to the posterior epiblast (Kimura-Yoshida et al., 2007; Perea-Gomez et al., 2001).

The expansion in the expression domain of Foxa2 could also account for the morphological changes in *Oct4* mutant embryos. Foxa2 has been proposed as a mesenchymal-to-epithelial transition (MET) driver in murine loss-of-function studies (Burtscher and Lickert, 2009) and in various human cancers (Myatt and Lam, 2007). Although it remains unclear whether Foxa2 possesses a gain-of-function phenotype in the embryo, it is possible that Oct4 limits the expression domain of Foxa2, which in turn controls epithelialisation.

Finally, the failure in our attempts to derive EpiSCs from *Oct4* mutant embryos is in agreement with published reports that EpiSCs can be derived from the postimplantation embryos as long as Oct4 protein is present and that EpiSC lines can be derived from anterior structures which would normally lack Oct4 only after its forced expression (Osorno et al., 2012).

**Fig. 8.**
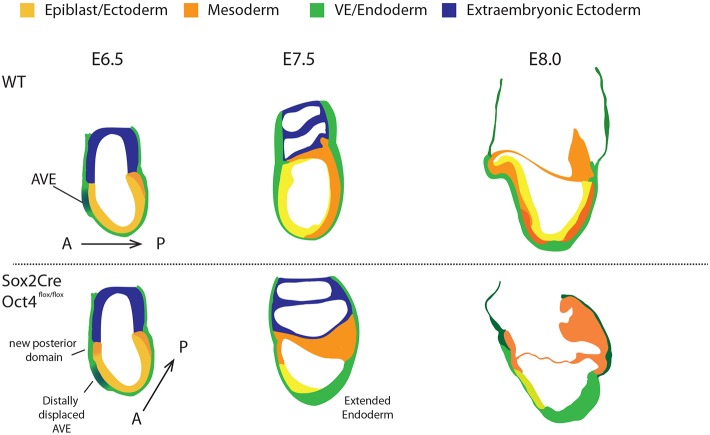
**Schematic representation of the phenotypes associated with conditional *Oct4* deletion.** Normal mouse peri-gastrulation development is depicted in the top panels. Below, the developmental progression of embryos lacking Oct4 from the onset of gastrulation is broken into three stages. At E6.5 the A-P axis is altered, resulting in distally displaced anterior visceral endoderm and appearance of a posterior-like domain in the proximal anterior region. Subsequently, the presumptive primitive streak region thickens with simultaneous failure to reduce E-cadherin and upregulation of definitive endoderm identity. Anterior structures fail to form and the embryo becomes disorganised.

Our results point towards a key role for Oct4 in orchestrating postimplantation development (Fig. 8). The data suggest there is a complex balance of identities in the epiblast, each competing with its neighbours and those shaping gene expression domains. In this context, Oct4 is likely to be playing a repressive role, in coordination with localised signalling pathways.

## MATERIALS AND METHODS

Experiments were performed in accordance with EU guidelines for the care and use of laboratory animals, and under the authority of appropriate UK governmental legislation. Use of animals in this project was approved by the Animal Welfare and Ethical Review Body for the University of Cambridge and relevant Home Office licences are in place.

### Mice and embryos

Homozygous mice in which the coding regions of both *Oct4* alleles (exons 2-5) are flanked by LoxP sites have been described previously (Le Bin et al., 2014). By mating female Oct4^fl/fl^ homozygous mice to male mice carrying Sox2Cre (Hayashi et al., 2002) and a single *Oct4*-null allele (Nichols et al., 1998), we established compound transgenic embryos using the scheme depicted in Fig. 1A. Mice and embryos were genotyped by PCR using validated primers as follows: Oct4LoxP, CTCAAACCCCAGGTGATCTTCAAAAC and GGATCCCATGCCCTCTTCTGGT; Oct4 null, GCCTTCCTCTATAGGTTGGGCTCCAACC (125N), GGGCTGACCGCTTCCTCGTGCTTTACG (122N) and GAGCTTATGATCTGATGTCCATCTCTGTGC (157N) (125N and 122N amplify the KO allele; 125N and 157N amplify the WT/Flox allele); Oct4LoxP recombined, ACTGAGAAGAAGGCAGCCTTAGC and GGATCCCATGCCCTCTTCTGGT; Cre transgene, GCGGTCTGGCAGTAAAAACTATC and GTGAAACAGCATTGCTGTCACTT.

Ear notches or embryos were added to 100 µl or 10 µl of digestion buffer, respectively [50 mM KCl, 10 mM TrisHCl (pH 8.3), 2.5 mM MgCl_2_, 0.1 mg/ml gelatin, 0.45% NP40, 0.45% Tween20] containing proteinase K (final concentration 200 µg/ml) and incubated at 55°C overnight. Proteinase K was heat inactivated at 95°C for 10 min. Ampliﬁcation was carried out on around 5 µl of DNA solution for 35 cycles (following a 95°C hot start for 10 min) of 94°C for 15 s, 60°C for 12 s, 72°C for 60 s, with a ﬁnal extension at 72°C for 10 min. Reaction products were resolved by agarose gel electrophoresis.

IOD mice homozygous for R26::CreERT2 (Vooijs et al., 2001) and floxed Oct4, used for the EpiSC-derivation and epiblast-outgrowth experiments have been described previously (Le Bin et al., 2014), and were maintained as homozygotes. To control for the risk that Cre activation by exogenous provision of tamoxifen is detrimental to epiblast outgrowth, we crossed the R26::CreERT2 homozygous mice to Confetti homozygotes (Snippert et al., 2010) to generate heterozygous embryos from which epiblasts were dissected.

Mice were maintained by in-house breeding on a lighting regime of 14 h light and 10 h darkness with food and water supplied *ad libitum*. Embryos were generated by natural mating. Detection of a copulation plug after pairing confirmed successful mating. Resulting embryos at noon on the day of the plug were considered to be at embryonic day (E) 0.5. All embryos used were isolated and dissected in M2 medium (Quinn et al., 1982).

### Embryo staining

Embryos were fixed in 4% PFA for 1 h at room temperature (RT), washed briefly with PBS and transferred to permeabilisation solution (PBS/0.25% Triton X100) for 1 h at RT. Embryos were blocked overnight at 4°C in blocking solution [PBS, 2% donkey serum (Sigma-Aldrich), 0.1% BSA (Sigma-Aldrich) and 0.01% Tween20 (Sigma-Aldrich)]. Primary antibodies (Table S1) were diluted in blocking solution at the concentrations stated and incubated overnight at 4°C. Embryos were washed three times, for at least 15 min each time in blocking solution, before incubating in Alexa Fluor-conjugated secondary antibodies (Molecular Probes) diluted 1:500 in blocking solution for 1 h at RT. Washes were repeated before mounting embryos in Vecta-shield (Vector Labs). Images were acquired using Leica TCS SP5 confocal microscope and processed with ImageJ. For paraffin embedding and H&E staining, standard protocols were used (Fischer et al., 2008). Embryos were orientated by mounting in 2% agarose in PBS prior to paraffin embedding. Antigen retrieval was performed by microwaving dewaxed slides in sodium citrate buffer [10 mM sodium citrate, 0.05% Tween 20 (pH 6.0)] for 20 min prior to immunostaining. X-Gal staining to reveal *lacZ* expression was performed following manufacturer's instructions (Sigma-Aldrich), based on the original embryo protocol (Beddington et al., 1989). Two to four embryos were stained for each combination of markers, and markers were stained in multiple combinations to validate results.

### Preparation of samples for RNA-sequencing

Embryos were manually dissected and the ExE removed. RNA extraction of whole-epiblast/VE samples was carried out using an Arcturus PicoPure RNA isolation kit according to the manufacturer's instructions. The ExE of each embryo was used for genotyping and processed as reported previously (Nichols et al., 1998) using primers listed above. Library preparation was carried out using the SmartSeq2 method (Picelli et al., 2014). Briefly, 1 ng of input material was used and PCR pre-amplification was performed with 15 cycles following polyA selection. Libraries were sequenced on a HiSeq4000, with paired end reads of 150 bp length each.

### Data alignment and analysis

Sequencing reads were processed to remove pre-amplification adapters with cutadapt 1.12 (Martin, 2011) and aligned to mouse genome GRCm38/mm10 with STAR 2.5.2a (Dobin et al., 2013). HTSeq-count (Anders et al., 2015) was used for reads quantification based on annotation from Ensembl 87. No significant differences were observed in read distribution (Fig. S5A), number of detected genes (Fig. S5B) or percentage of mapped reads (Fig. S5C) across samples.

Data analysis was performed in R. Aligned reads were converted to FPKM. Differential gene expression analysis was performed with DESeq2 (Love et al., 2014) using an adjusted *P*-value threshold of 0.05. To correlate the changes in gene expression upon *Oct4* deletion with the 3D map of the gastrulating mouse embryo, we used the ‘Zipcode Mapping’ feature of iTranscriptome (Chen et al., 2017; Peng et al., 2016). GAGE (Luo et al., 2009) was used for pathway and gene ontology (GO) analysis on the differentially expressed genes but revealed no significant enrichment (not shown). PathVisio (Kutmon et al., 2015; van Iersel et al., 2008) was used to visualise gene expression of signalling pathway components. Pathways were adapted from WikiPathways templates (Kelder et al., 2012; Kutmon et al., 2015). Data have been deposited in GEO under accession number GSE103403. Oct4-binding peaks were obtained from Matsuda et al. (2017) (GSE74636). A merged table of gene expression level, differential analysis and nearby Oct4 binding peaks is presented in Table S2. To investigate individual genes, bigWig files were uploaded onto the UCSC Genome browser (https://genome.ucsc.edu/).

### Epiblast outgrowths

E6.5 embryos were explanted on plates coated with fibronectin in basal N2B27 medium (made in-house) lacking exogenous signalling components or cytokines. Explants were cultured for 48-72 h, fixed and processed for E-cadherin immunohistochemistry. For the differentiation to cardiomyocytes, E7.5 embryonic tissues were dissected free from extra-embryonic tissues and plated on a feeder layer of mitotically inactivated murine embryonic fibroblasts in basal N2B27 medium and cultured for 48 h before imaging.

### *In vitro* differentiation of tamoxifen-inducible Oct4-deleted cells

The protocol was adapted from Morgani et al. (2018). IOD lines were routinely passaged in 2i/LIF medium in N2B27. For differentiation assays, IODs were directly plated in EpiLC media as previously described (Hayashi et al., 2002). Briefly, 2.5×10^4^ cells/cm^2^ were plated on to fibronectin coated plates in N2B27 medium supplemented with activin A (20 ng/ml), FGF2 (12.5 ng/ml) and 1% KSR. After 48 h, cells were split with accutase and 8.0×10^5^ cells plated directly in EpiLC medium supplemented with 10 µM ROCK inhibitor (Y27632, Merck) onto micropatterned substrates (12 well plate). The inhibitor was not necessary for survival, but it allowed rapid cell attachment to the micropatterns as a monolayer. After 2 h, the non-attached cells were removed by washing the wells twice with DMEM/F12+0.1% BSA before changing the medium to fresh EpiLC medium without ROCK inhibitor.

### Micropattern fabrication and stamping

Micropatterns were produced by using a microcontact printing protocol, adapted from Thery and Piel (2009). A mould was fabricated by photolithography. The negative photoresist SU-8 2100 (Microchem) was spin-coated on to a silicon wafer at a speed of 505 ***g*** then baked as per manufacturer's instructions to achieve a coating of 100 µm. A photomask (JD Phototools) containing an array of circles of 1000 µm diameter was used to selectively expose the photoresist to UV radiation (10 mW/cm^2^) for 26 s. Following post-exposure bake, as per the manufacturer's protocol, PGMEA (propylene glycol methyl ether acetate) was used to develop unexposed regions of photoresist. De-gassed 9:1 (base:curing agent) polydimethylsiloxane (PDMS) was poured into the container containing the mould and cured at 60°C for 90 min.

PDMS stamps were cut to fit 12-well plates. Stamps were washed once with 70% ethanol, before rinsing with distilled water and allowing them to dry. To make them hydrophilic, stamps were subjected to plasma cleaning for 20 s. Immediately, a solution of 100 µg/ml laminin in PBS was added on top to ink the stamps. Stamps were incubated for at least 3 h at RT with the laminin solution. To generate the micropatterns, the laminin was aspirated and the stamps allowed to air dry. Twelve-well plates were plasma treated for 2 min before adding the stamps and applying pressure to print the pattern. The stamps were removed from the wells after 10 min. The wells were covered with a 0.2% solution of pluronic F-127 in PBS (Sigma Aldrich) to render the non-patterned surface of the well hydrophobic. After 30 min, the pluronic F-127 solution was aspirated and the wells washed three times with PBS (10 min each wash) to remove excess coating. Micropatterned wells were stored in PBS and used later in the day (longer storage not tested).

### EpiSC derivation

Embryos were dissected at E5.75 and E6.5. The epiblast was manually separated from the Reichert's membrane, VE and ExE. Whole epiblasts were plated on fibronectin-coated plates in pre-equilibrated N2B27 medium supplemented with 20 ng/ml activin A, 12 ng/ml FGF2 and 10 µM XAV929 (Sigma), as reported previously (Sumi et al., 2013). Outgrowths were passaged after 5 days.

### Image analysis

Quantification of immunofluorescence was performed using ImageJ. Briefly, nuclei were designated as regions of interest (ROIs) on the DAPI channel, from the anterior to the posterior end of the epiblast. The intensity of the ROIs in the other channels was quantified and the values plotted relative to cell position. For each staining, representative images and corresponding quantification is provided. To quantify the E-cadherin intensity at the membrane, raw intensity measures were obtained by measuring intensity across a line (plot profile, ImageJ). Using a custom MATLAB code, the line profile was low-pass filtered (code courtesy of Kevin Chalut, Wellcome-MRC Cambridge Stem Cell Institute, UK) and we calculated the staining intensity at the membrane as the difference between the major peak and subsequent trough along the line. To quantify differentiation on micropatterns, all channels were thresholded based on unstained controls. The area of positive staining was calculated for each channel and shown as a fraction of the area covered by DAPI.

## Supplementary Material

Supplementary information
