# Supplementary material for: Oct4 regulates the embryonic axis and coordinates exit from pluripotency and germ layer specification in the mouse embryo
Source: Development. 2018 Jun 18;145(12):dev159103. doi: 10.1242/dev.159103 (PMC6031404; doi:10.1242/dev.159103)
Supplement: Supplementary information [file develop-145-159103-s1.pdf]

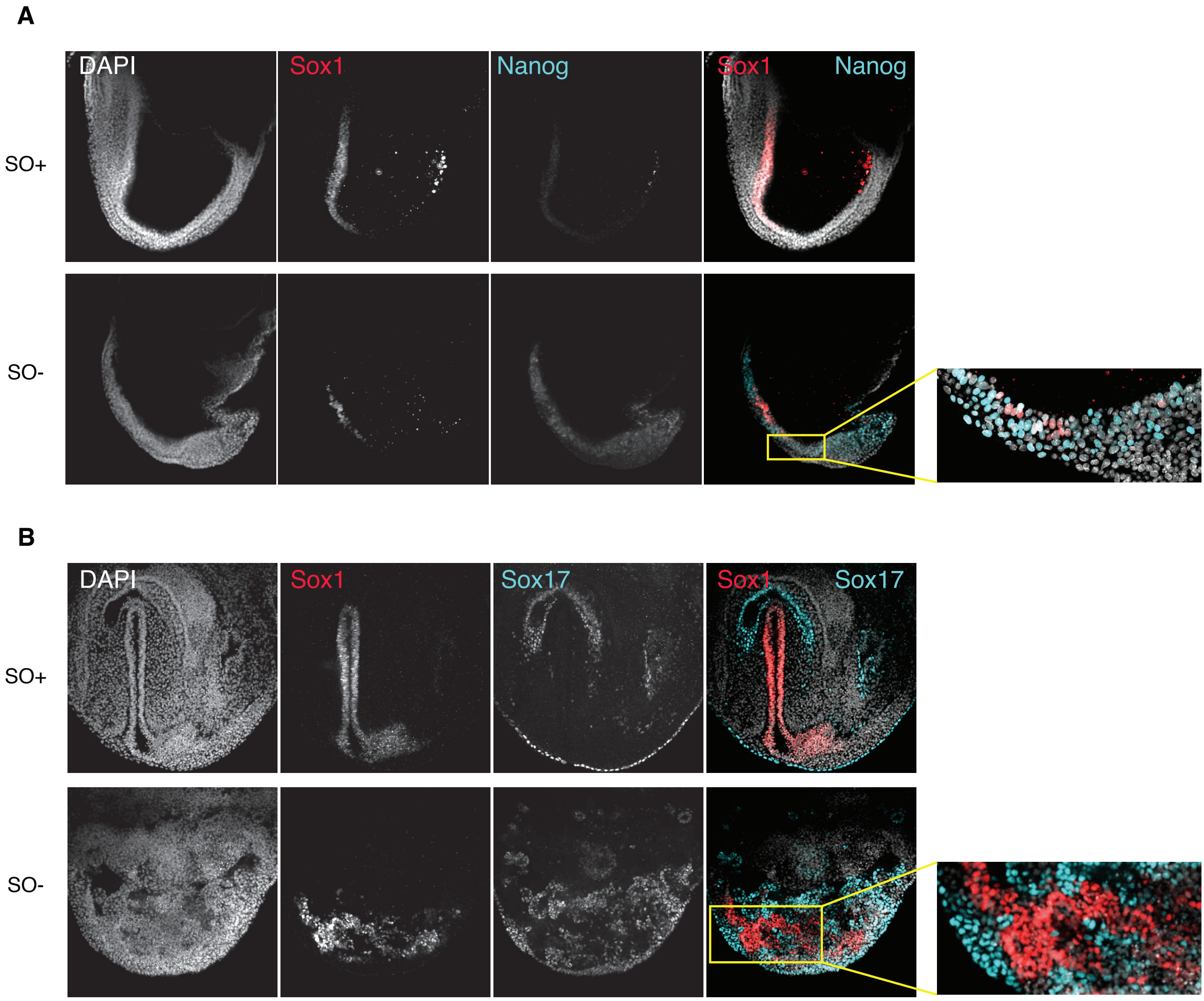

Fig. S1. Oct4 mutant embryos do not aberrantly co-express lineage determinants.

Immunostaining of E7.5-7.75 embryos for (A) Sox1 (red) and Nanog (turquoise) and (B) Sox1 (red) and Sox17 (turquoise). Top row of panels in each case shows SO + embryos and bottom panels show SO- Oct4 mutants. Insets highlight regions in mutant embryos where the domains of marker expression have become disrupted, but there is apparently no co-expression of distinct lineages within any cell (A 40X, B digital zoom).

Figure S2

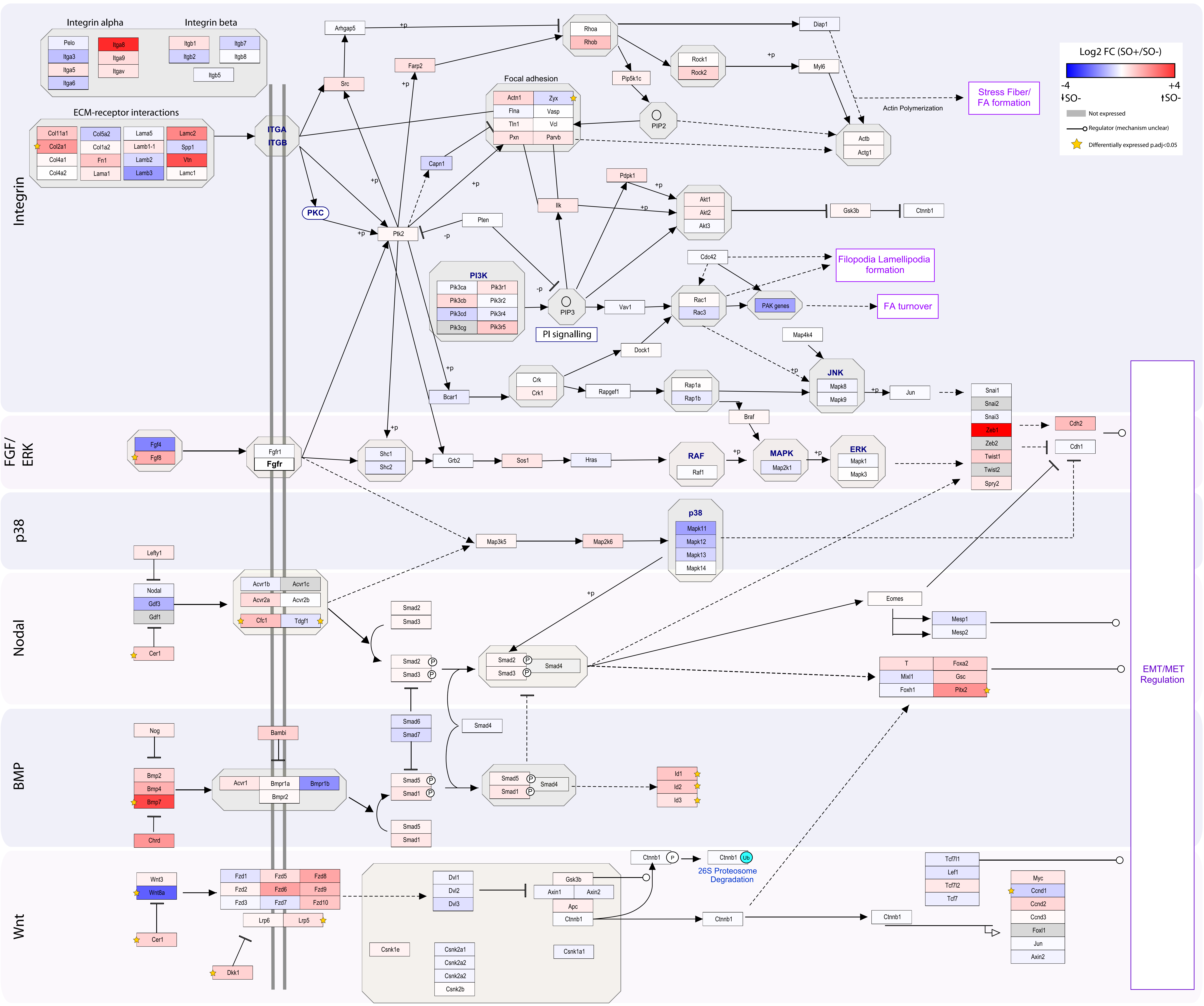

Fig. S2. Signalling pathway components relevant to cell fate decision and gastrulation in the post-implantation embryo. Only detected genes are shown. Each component is coloured based on the log2FC(SO+/SO-). Stars indicate differentially expressed genes (p.adj<0.05).

Figure S3

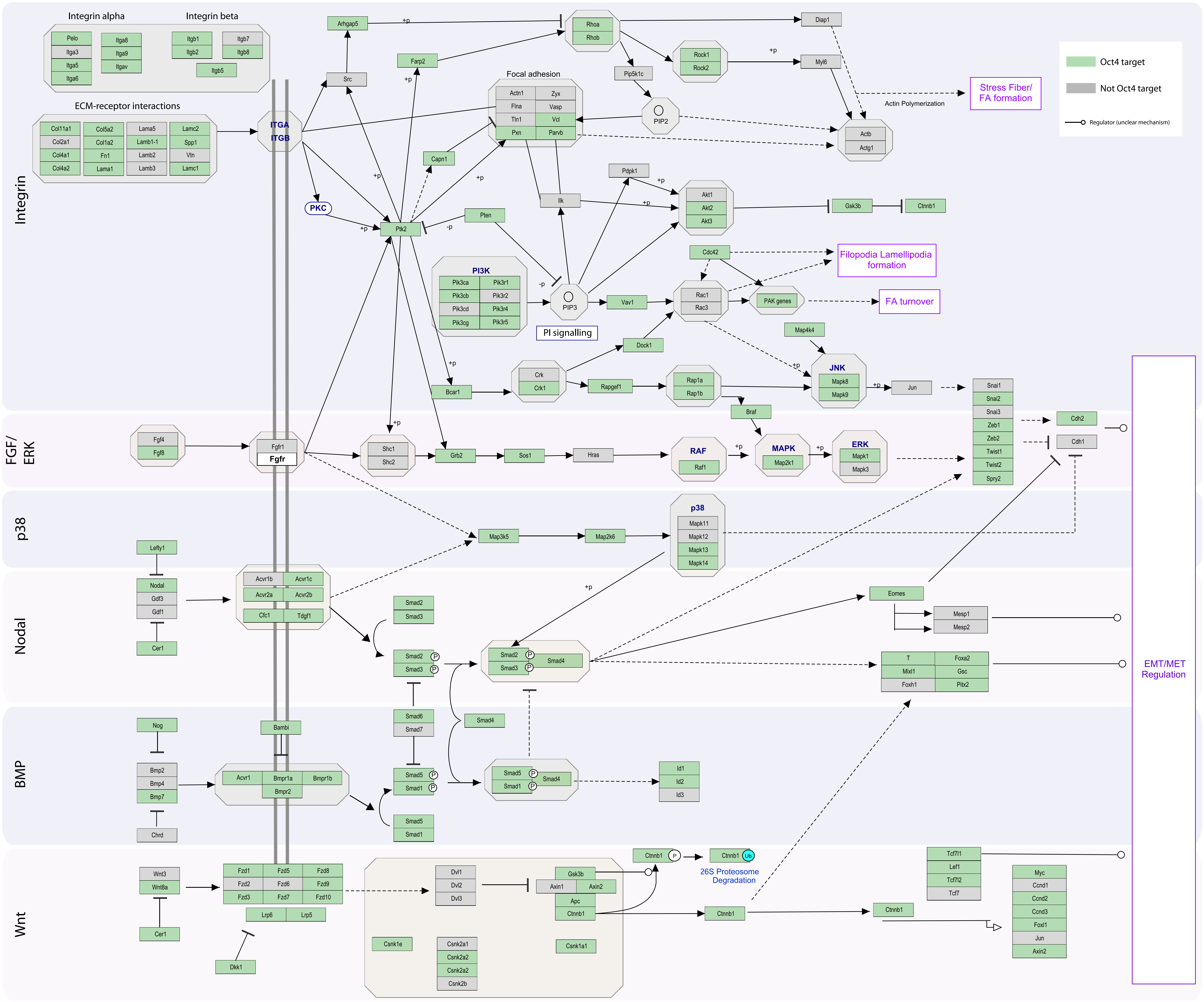

Fig. S3. Signalling pathway components relevant to cell fate decision and gastrulation in the post-implantation embryo. Genes shown in green have Oct4 binding peaks in EpiSCs within 50kb of the transcription start site. Chip-seq data from (Matsuda et al., 2017).

Development • Supplementary information

Figure S4

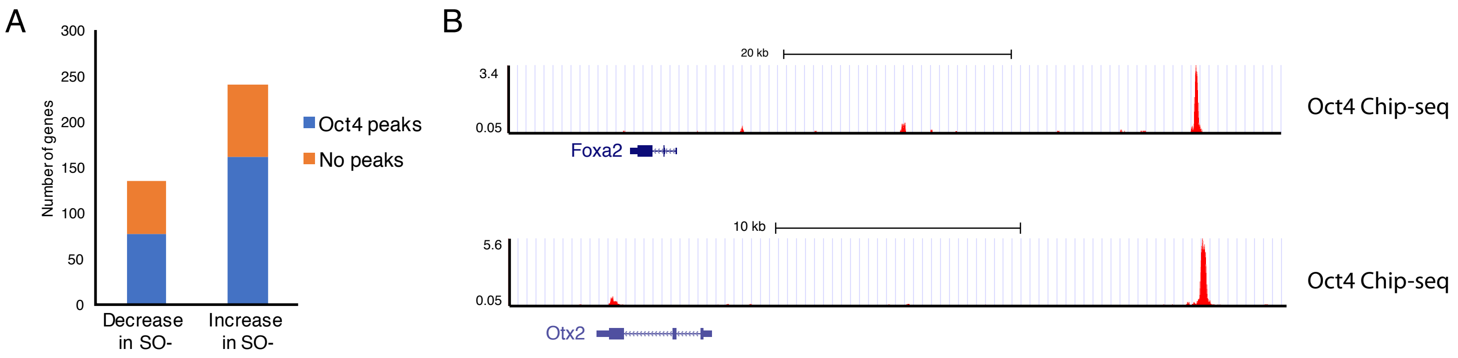

Fig. S4. (A) Breakdown of the number of genes upregulated and downregulated in SO- embryos compared to SO+ embryos and proportion of Oct4 bound genes. (B) Individual Chip-Seq tracks showing Oct4 binding within 40kb and 20kb of the promoter of Foxa2 and Otx2 in EpiSC.

Development • Supplementary information

Figure S5

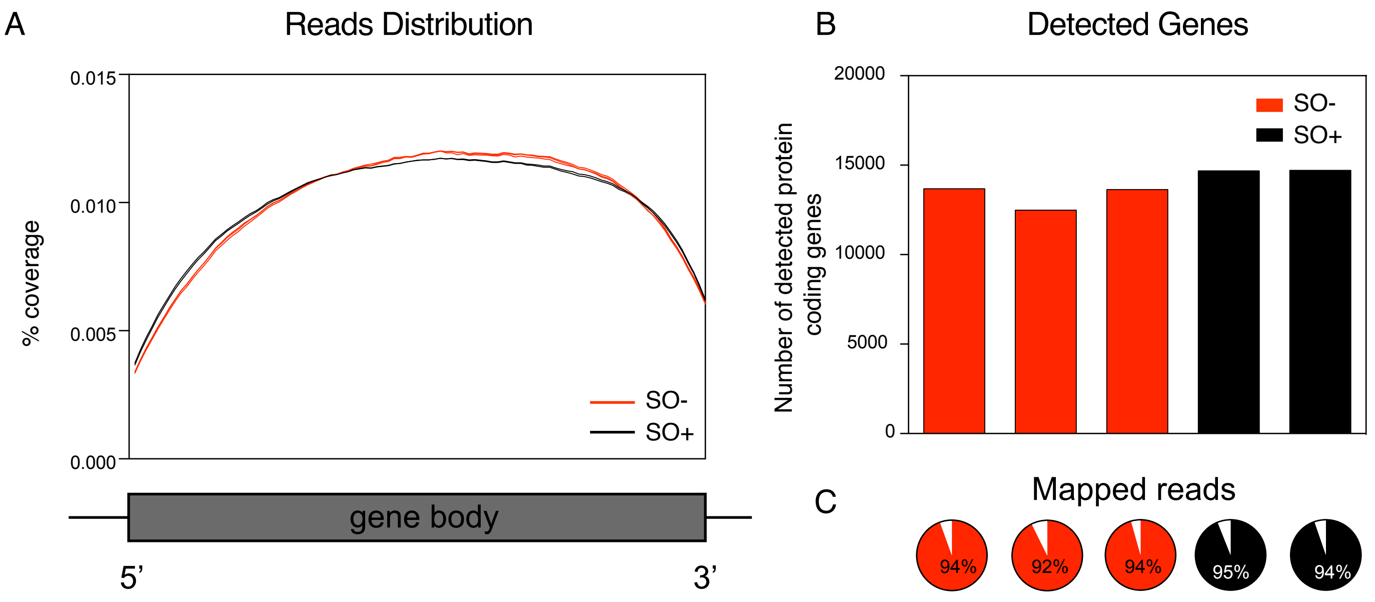

Fig. S5. RNA-seq data quality control. (A) Read distribution, (B) number of detected genes and (C) percentage of mapped reads across RNA-seq samples.

Development • Supplementary information

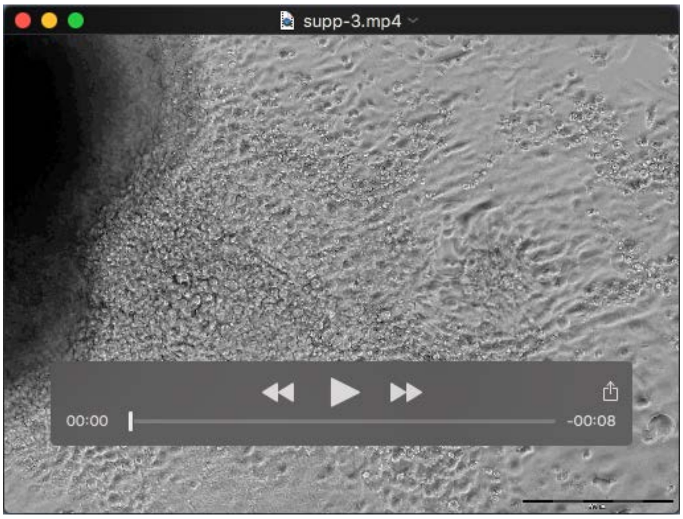

Movie S1. Live imaging of SO- outgrows showing beating cells.

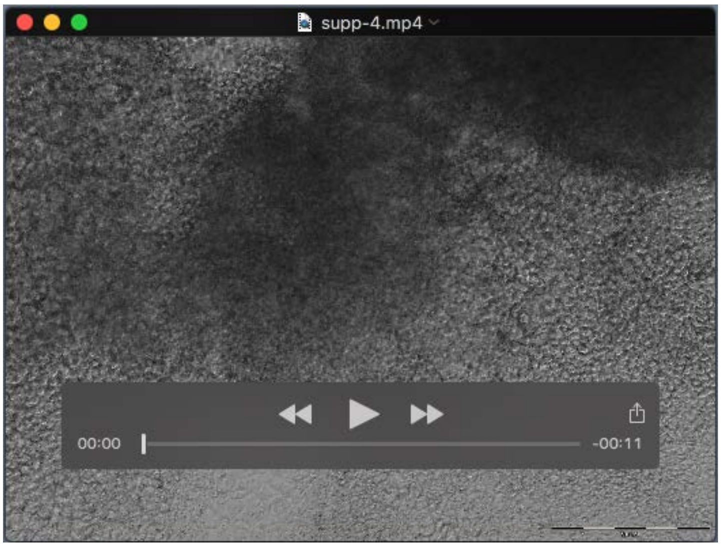

Movie S2. Live imaging of SO+ outgrows showing beating cells.

Development • Supplementary information

Table S1. Antibodies used in this study

Antigen	Company	Cat. No	Dilution
Oct4	Santa Cruz	SC-5279 (C-10)	1:200
Oct4	Santa Cruz	SC-8628 (N-19)	1:200
Nanog	eBiosience	eBioMLC-51	1:200
Sox2	Abcam	ab97959	1:200
Brachyury	R&D	AF2085	1:200
Mixl1	Elephanty's group		1:200
Foxa2	Santa Cruz	SC-6554 (M-20)	1:200
Foxa2	Cell Signalling	3148	1:200
E-cadherin	Abcam	ab15148	1:200
Sox17	R&D	AF1924	1:200
Otx2	Abcam	ab21990	1:200
Sox1	Cell Signalling	4194	1:200
Tbx6	R&D	AF4744	1:200
Cer1	R&D	MAB1986	1:100
Snail	Cell Signalling	3879	1:100

Table S2. Gene expression analysis of SO+ and SO– embryos.

Click here to Download Table S2

Development • Supplementary information
